# Improving communication of the concept of 'treat-to target' in childhood lupus: a public and patient (PPI) engagement project involving children and young people

**DOI:** 10.1186/s41927-022-00300-z

**Published:** 2022-10-15

**Authors:** R. S. Elliott, E. Taylor, J. Ainsworth, J. Preston, E. M. D. S. Smith

**Affiliations:** 1 School of Medicine, University of Liverpool, Liverpool, UK; 2 Institute of Life Course and Medical Sciences, University of Liverpool, Liverpool, UK; 3 Department of Paediatric Rheumatology, Institute in the Park, Alder Hey Children’s NHS Foundation Trust, Liverpool, UK

**Keywords:** Treat-to-target, Lupus, Paediatric rheumatology, Patient and public involvement

## Abstract

**Background:**

A treat-to-target (T2T) approach, where treatment is escalated until a specific target is achieved, and re-escalated if the target is lost, has been proposed as a strategy to improve Childhood Systemic Lupus Erythematosus (cSLE) outcomes. Previous studies involving children and young people (CYP) have identified that the concept of T2T can be difficult to understand by CYP and their families. We aimed to explore the views of CYP participating in existing public and patient involvement (PPI) groups in relation to a proposed animation that is being developed to explain the concept of T2T to CYP who will be eligible for a future cSLE T2T trial.

**Methods:**

An illustrated animation storyboard was developed on PowerPoint, to be used alongside a contemporaneous voiceover to simulate the animation for CYP participating in three existing CYP PPI groups (GenerationR, Lupus UK, and YOUR RHEUM). Mixed methods were used to generate CYP feedback on the resource, including on-line surveys and qualitative topic-guided discussion, noting CYP suggestions for improvement. Changes were made iteratively to the resources. Pre/post workshop questionnaires to assess the impact of the resource on their understanding of T2T were completed anonymously.

**Results:**

40 CYP were consulted; 16/40 (40%) from GenerationR (median age 15-years [IQR 12–15]), 12/40 (30%) from Lupus UK (median age 27-years [IQR 22–30]), and 12/40 (30%) from YOUR RHEUM (median age 17-years [IQR 16–21]). 62% of respondents had an underlying rheumatic condition. Pre-workshop median participant understanding of T2T was 2/10 [IQR 1–4], on a 1–10 scale (1 = “no understanding at all”, 10 = “completely confident in my understanding”). After viewing the resource, participant understanding improved to a median of 9/10 [IQR 8–10], *p* < 0.0001). Overall, participants felt that the animation greatly improved their understanding of the concept of T2T, making several suggestions for improvement.

**Conclusion:**

Involvement of CYP in research is crucial to help improve the design/delivery of studies, ensuring relevance to CYP and their families. This manuscript demonstrates the involvement of CYP in the development of an animation that will be integral to a future clinical trial, helping to describe the T2T approach in a comprehensible way to eligible CYP and their families, supporting study recruitment.

**Supplementary Information:**

The online version contains supplementary material available at 10.1186/s41927-022-00300-z.

## Background

Childhood onset systemic lupus erythematosus (cSLE, otherwise known as Juvenile Systemic Lupus Erythematosus, JSLE) is a chronic, multisystem autoimmune/autoinflammatory disorder [1]. It is in many ways more aggressive than adult-onset SLE, with higher disease activity, medication burden/toxicity, and more severe internal organ involvement (e.g. lupus nephritis, neuropsychiatric or haematological manifestations) [1–4]. Despite survival significantly improving over the past 50 years, [5] mortality rates remain higher in lupus as compared to the general population; and are also much higher in cSLE versus adult SLE [6]. CSLE patients are at particular risk of developing permanent organ damage early in the disease course, [7] and display lower health-related quality of life (HRQOL) than healthy children and young people [8].

‘Treat to target’ (T2T) involves adjustment or escalation of treatment until a specific pre-defined target is achieved, and re-escalation of treatment if the target is lost. This target based treatment paradigm is now part of routine clinical care in many areas of medicine (e.g. rheumatoid arthritis, hypertension, diabetes) [9]. Internationally, there is great enthusiasm for the development of T2T in cSLE [10–12] and adult-onset SLE [13–15]. It is envisaged that the cSLE T2T approach will enable use of existing treatments in a more structured way, aggressively controlling disease activity earlier in the disease course, preventing accrual of organ damage, and crucially improving HRQOL [10].

The TARGET LUPUS research programme (Targeting disease, Agreeing Recommendations and reducing Glucocorticoids through Effective Treatment in LUPUS) has been established to facilitate development of a T2T clinical trial for cSLE [10, 11]. A UK multicentre qualitative study undertaken as part of the TARGET LUPUS research programme showed that participants had difficulty understanding the concept of T2T [10]. Such difficulty could hinder informed consent and consequently reduce the number of patients willing to participate in a T2T study. It is difficult for patients to find information on T2T by themselves, as most available resources are aimed at medical professionals. Audio-visual resources are particularly useful for teaching patients with low health literacy [16] and they have been used successfully to explain the concept of T2T to rheumatoid arthritis patients [17].

This PPI initiative aimed to explore the views of CYP in relation to a proposed animation that will be used in the future to explain the concept of T2T to CYP with cSLE who are eligible for a T2T clinical trial.

## Methods

### Study design

Mixed methods study including on-line surveys and qualitative topic-guided discussion to generate CYP feedback on a proposed animation that will be used to explain the concept of T2T to CYP with cSLE who are eligible for a T2T clinical trial.

#### Storyboard

The storyboard for the animation was developed as a PowerPoint presentation with an accompanying real-time voiceover to explain the concept of T2T to CYP. The storyboard showed the patient journey through T2T appointments, illustrating what this would entail for the patient and family.

#### PPI groups

Inclusion/exclusion criteria used to identify suitable PPI groups were as follows: (1) existing young people’s PPI groups predominantly including young people under the age of 25 years, and (2) groups including CYP who had not previously been part of a T2T study. Groups were excluded if (1) their participants had previously participated in a T2T study, or (2) they had previously discussed the concept of T2T with researchers. Three existing CYP PPI groups meeting these inclusion/exclusion criteria were identified and consulted as part of this initiative [GenerationR Liverpool Young Persons Advisory Group (YPAG), Lupus UK, YOUR RHEUM YPAG]. GenerationR Liverpool YPAG is a well-established advisory group set up in 2006 to support the design and delivery of clinical paediatric research. The Liverpool YPAG is one of many YPAGs across the UK who form the GenerationR Alliance coordinated by the National Institute for Health Research (NIHR) Alder Hey Clinical Research Facility PPI team [18]. Lupus UK is a national charity that provides support for children and adults with SLE. It holds monthly meetings which provide peer support for patients and are frequently attended by researchers undertaking PPI activities [19]. YOUR RHEUM YPAG (also part of GenerationR Alliance) is a group for 11–24 year olds across the UK with diagnosed rheumatic conditions to advise, input and shape adolescent and young adult rheumatology research [20, 21]. A series of three virtual meetings was undertaken with each PPI group between January 2021 and March 2021.

### Structure of the PPI group meetings

Meetings with each organisation were held over Zoom and began with an anonymous pre-session survey to rank their understanding of T2T on a scale of 1–10, where 1 = “no understanding at all”, 10 = “completely confident in my understanding”. Demographic information was collected anonymously, including ethnicity, age, gender, and which health condition(s) they suffered from. The storyboard animation was then simulated for participants using the PowerPoint and the simultaneous voiceover. Facilitated discussion followed, exploring the view of the participants on (a) how to clearly communicate the difference between T2T and standard care, (b) aspects of the proposed animation that they liked, (c) aspects that they felt could be improved, (d) terminology that they found unclear or confusing, (e) other suggestions. At the end of the meeting, participants re-ranked their understanding of T2T on a scale of 1–10 through an anonymised post-session survey.

The meeting co-ordinators met after each PPI meeting to discuss their notes, reflect on the CYP suggestions, and iteratively made changes to the animation and associated voiceover to address the points raised by the CYP.

### Analyses

Meeting notes were reviewed after each meeting. Common themes and key quotes were used to make changes to the animation and associated voiceover. Information collected from pre and post surveys was assessed to determine if the resources clarified the concept of T2T. Wilcoxon matched-pairs signed rank test was used to compare pre and post meeting scores relating to understanding of T2T (1–10 scale). After all changes were applied, the resource was animated with the help of the University of Liverpool animation team. NHS ethical approval was not required for this PPI initiative as this was a consultation exercise in research design [22, 23]. The NHS Health Research Authority differentiates between research consultation where individuals are not research participants but acting as specialist advisors, versus research participation where individuals are the subjects being researched [22, 23].

## Results

### Participants

Our results are based upon three PPI group meetings involving 40 CYP; 16/40 from GenerationR Liverpool YPAG (median age 15-years, IQR 12–15), 12/40 from Lupus UK (median age 27-years (IQR 22–30), and 12/40 from YOUR RHEUM YPAG (median age 17-years, IQR 16–21). 32/40 (80%) of participants were female and 8/40 (20%) were male. Across the three CYP PPI groups, 15 participants had SLE, eight had Juvenile Idiopathic Arthritis (JIA), two had Asthma, one participant had Mixed Connective Tissue Disease (MCTD) and one suffered from Migraines. The remaining 13 participants did not suffer from any health conditions. Each PPI group meeting lasted between 60–90 min. 39/40 (98%) participants completed the pre-session survey, 37/40 (93%) completed the post session survey.

### Pre-session survey

Participants were asked *‘Do you know what treat to target is?’*. 27/39 (69.2%) participants answered, ‘*No I don’t know what it is’*, 10/39 (25.6%) responded with ‘*I’m familiar but don’t completely understand it’* and 2/39 (5.1%) of responded with ‘*Yes I completely understand the concept’*. On a 1–10 scale (1 = “no understanding at all”, 10 = “completely confident in my understanding”), the median pre-session understanding of T2T was 2 [IQR 1–4].

### How to clearly communicate the difference between T2T and standard care

**Fig. 1:**
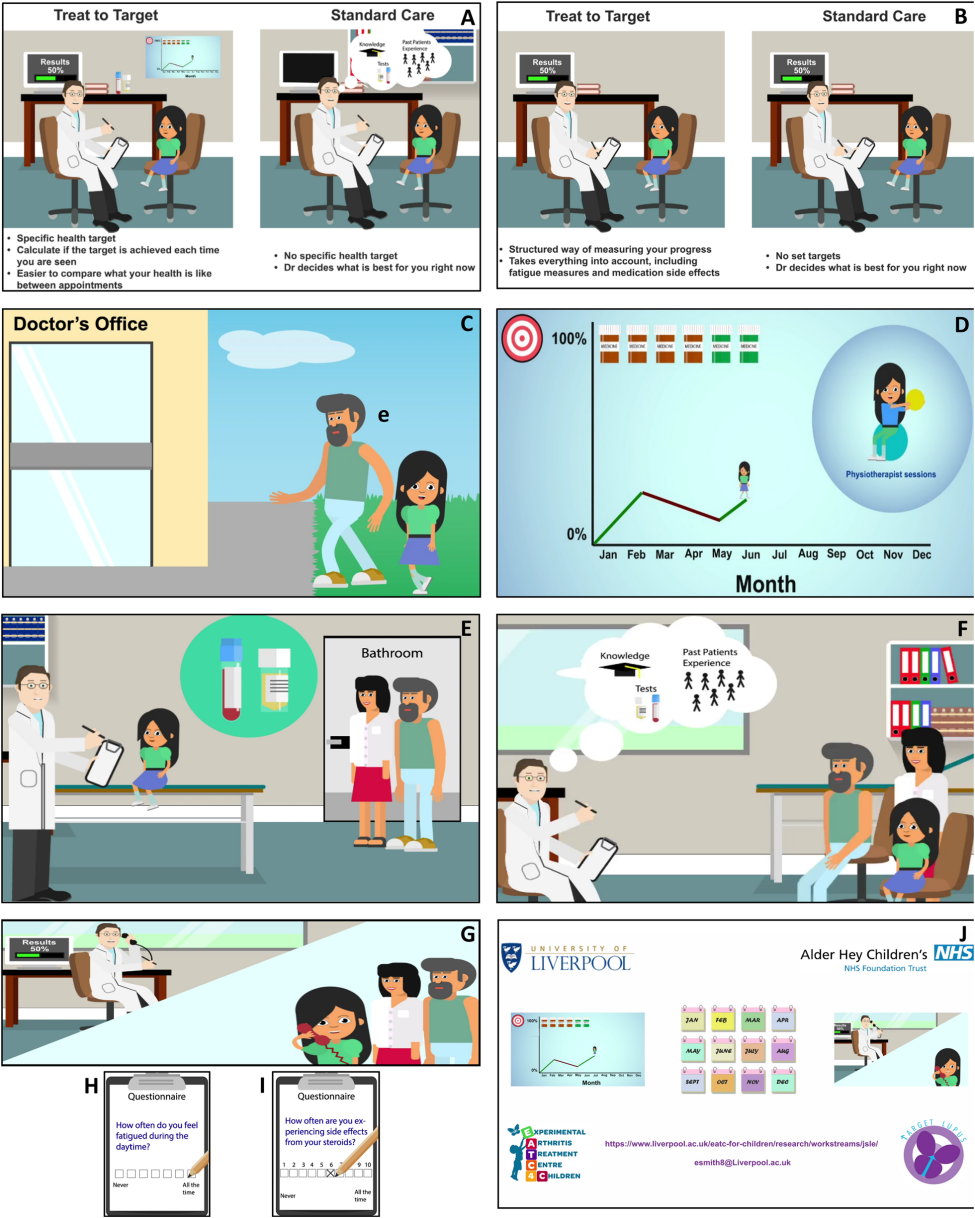
Animation stills. **A** + **B** Side-by-side comparison of T2T and standard care, **C** Lucy, the character participating in the T2T cSLE study with parent, **D** Graph demonstrating continual monitoring towards Lucy’s target. Lucy’s progress is initially good but then her condition declines and new treatment and physiotherapy input is recommend which helps Lucy start to progress again towards her target, **E** The doctor is patient focused, with parents in the background for support, **F** + **G** The doctor uses his knowledge, experiences with past patients, Lucy’s test results, knowledge of whether Lucy’s target has been reached and the results of the patient reported outcome measures (assessing health related quality of life, fatigue and steroid toxicity) to select the best treatment approach for Lucy, **H** + **I** Patient reported outcome measures to assess steroid toxicity and fatigue and support the structured assessment of Lucy’s Lupus, **J** Informational slide providing further contacts for CYP interested in participating in a T2T cSLE study. The images depicted within this figure are the authors own and are taken directly from the animation

When shown the initial version of the storyboard, some CYP found it difficult to completely understand what the differences were between T2T and standard care was: *“I was slightly confused at how it was different [T2T vs standard care] just because (…) my experience of health is that it is tailored to you, you go to your doctor if it’s not working, and they will speak to you and work it out”*. The CYP suggested adding a clear ‘side-by-side’ comparison of T2T and standard care. This change was applied to the storyboard (See Fig. 1A + B) improving understanding of the difference between T2T and standard care within subsequent PPI groups: *“I thought it was good [the animation storyboard], I had a few questions come up during the presentation, but then they were answered later, even specifically one question I had was directly answered later on. It was ‘how is this different to how your doctor already treats you?”* and *“Yeah definitely [the difference between T2T and standard care was clear], the summary at the end really helped”.*

#### Aspects of the proposed animation that were liked by the CYP

The storyboard followed a cSLE patient called ‘Lucy’ who was participating in a T2T study (See Fig. 1C). This CYP liked this approach: *“I liked the character—it made it more personal”.* The PPI groups were familiar with the uses of leaflets to explain research studies but felt that an animation would be easier to understand and demonstrate what the study would involve for the patient: *“It’s always paper information when I’ve been informed of studies in the past, for example, when I was 12 and I didn’t understand it. Animation is better, a really good idea.”*

During the course of Lucy’s involvement in the T2T study her SLE starts to flare, this was described by her experiencing headaches, joint pain, extreme tiredness and being unable to participate in physical education (PE). A number of CYP felt that taking part in PE in school was an effective example of the disease symptoms affecting real life *“I think it is a good practical one because when I was younger, if I was really ill, those were the days I couldn’t join in. Not being able to do PE is kind of like a marker because it’s something that you have to do every wee*k”.

Within the storyboard, a graph was used to demonstrate visually that over-time there was continual monitoring of Lucy’s target. During the initial part of the study she made good progress towards her target but then her condition started to worsen leading to a prompt change in treatment/referral to a physiotherapist, allowing Lucy to get back on track towards attainment of her target (See Fig. 1D). The participants felt that this was a good was to illustrate how treatment would be adjusted in response to the patients progress towards the target: *“The graph is a nice, visual way to look at progress and think about where you’re heading”* and *“The patient and the family need to know if it isn’t getting better…you’d rather know that not…so I quite liked the numbers…I think transparency is really important”.* However, some participants were concerned that this could be daunting for the patient: *“What happens if it [the graph] goes down? If I could see I was further away from the target, that would scare me”*, and therefore recommended that use of the graph in discussions with the patient/family should be considered an individual decision.

#### Aspects that the CYP felt could be improved

The original voiceover only included a single narrator, but the CYP suggested that including more characters would improve the animation: *“Double act would be good [of both patient and doctor speaking in the voiceover]—mix of personal and professional.”* Including both a patient and parental voice could also highlight the importance of parents supporting their children to be involved in research, whilst continuing to emphasise that the overall focus should remain on the young person as an active participant in the research and related decision making: *“I definitely think add in a parent [to the animation to attend appointments with the child character] but don’t have them as a huge part like it's everything about them (…) I’ve always felt centre of my treatment and the doctor is speaking to me rather than my mum, even though I might not always understand everything. Having a parent there is nice to reassure a child” and “I would say maybe take the speech bubble off the parent just so it shows the doctor is speaking to the child”.* (See Fig. 1E).

For certain aspects of the animation, participants felt that they would be explained better by a doctor character *“I think people would prefer a healthcare professional because if you hear it from a healthcare professional who’s had loads of experience it sounds like they know completely exactly what they are doing”.* Within the final storyboard inclusion of a range of characters greatly improved engagement for the CYP in the storylin*e “I really loved the storyline and it made it more interesting having different voices. It keeps you more engaged having different people speak. It felt more relatable, like you could see it from the patient’s perspective”* and *“I agree, I think it was more engaging that way, I think it was great”.*

The participants disliked the phrase *‘If you aren’t meeting your target’* as they felt it placed an element of accountability on the patient, rather than the treatment being at fault and unsuccessful: *“The use of the phrase “if you aren’t meeting your target” bothers me a little. It’s not the patient’s fault and it is not their ownership that they aren’t meeting their target”.* In response to this the terminology used by the doctor in the voiceover was changed to *“It looks like things haven’t improved since your last visit and may be a bit worse”* removing any mention of not meeting *“your target”,* with the doctor going on to say *“we need more help to meet your target”* taking the onus away from the patient and highlighting that the treatment is responsible for whether the target is reached, not the patient.

#### Terminology/wording that required refinement

The original storyboard used the words ‘extreme tiredness’ as a lay way to explain fatigue. The CYP with Lupus in particular preferred the wording of ‘fatigue’ as opposed to ‘tired’ when describing SLE symptoms, although they acknowledged that the word ‘tired’ would be easier for younger children to understand: *“I was thinking fatigue for the same reason…I really think they’re two different things”* and *“[I] Struggled with fatigue for years, doctors note it but nothing is done, it was always on the symptom list but didn’t go away. Everyone has suffered with it at some point, no one seems to know how to deal with it or how we can help ourselves”*.

The original storyboard showed a computer calculating whether the target had been attained or not and therefore guiding treatment. This was concerning to some CYP who wanted to be sure that the doctor would still be the one treating the patient, guided by the computer but also taking into account their professional knowledge (See Fig. 1, F + G): *“It just feels to me where you talk about the computer doing the number crunching and all the rest, it’s all about the computer treating me it’s not about my doctor treating me…although we love the fact that he’ll have access to a huge database of information on a computer that will help guide him. At the end of the day, it’s our doctor who should be treating us.”* This was addressed by adding the following to the voiceover: *“the doctor will assess if the target has been fully met”* and “*with normal care there is no set target, treatment changes are based on the doctors opinion at the time of the appointment, with treat to target, the doctor takes into account fatigue, medication side-effects and how Lupus affects your day to day life (using structured assessments). With normal care this is something your doctor may or may not assess”* (Additional file 1).

#### Other thoughts on T2T

Overall, the CYP thought that T2T sounded like a good way to structure care: *“I think it’s really good, I like the idea and the concept myself (…) it is easier to kind of understand the concept of T2T and I suppose give anybody who’s going into that situation more clarity on what they’re going to be getting I guess from the doctor.”* The animation showed Lucy and her doctor discussing and agreeing what her individualised target should be. The CYP liked this approach: *“The ability to set different targets is great, all patients experience Lupus differently so different goals, the ability to personalise is good.”* Use of structured questionnaires to measure fatigue and steroid toxicity was also viewed as a benefit of T2T, as many felt that this is overlooked by their doctors (See Fig. 1H + I): “*I really like the idea of a fatigue measure, I’ve never had any questionnaire on it or similar and yet it’s one of the most debilitating parts of day-to-day life, it would be really useful to me to see how it’s changed for me over the years compared to at the moment” and* “*I think the steroid toxicity measure is a great idea; as a person who has been on steroids for over 2 years it is a concern.”*

**Fig. 2:**
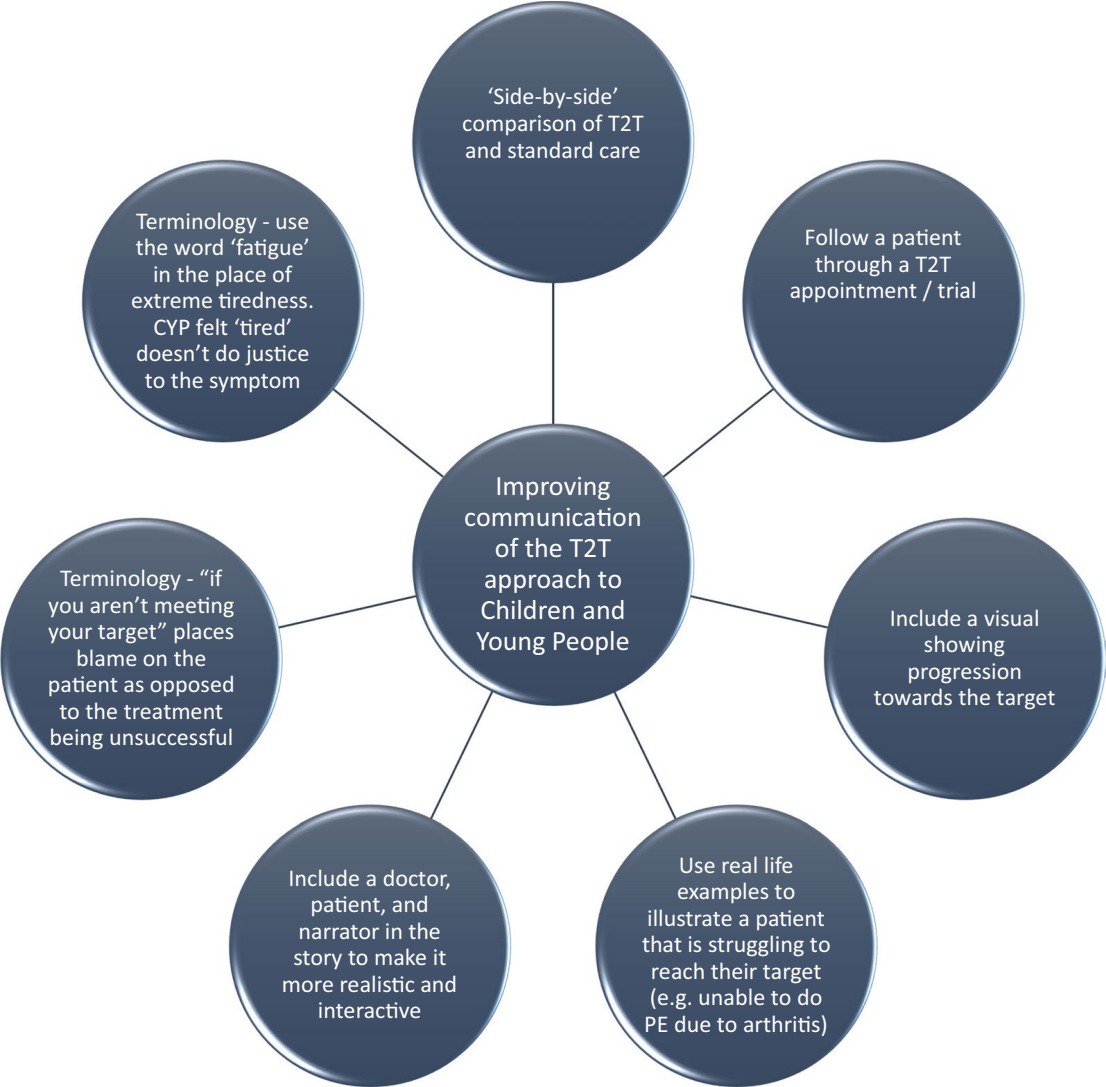
Summary of the study findings. CYP = children and young people. PE = physical education

Some CYP, particularly those who lived far away from their hospital were concerned about the impact that a T2T approach would have on the patients life, in view of the suggestion that a T2T approach would include more frequent hospital appointments: *“The time thing needs to be made clearer (…) my hospital is quite far away so appointments every six weeks I would hear that and think oh god.”* This led to discussion of the use of phone/video consultations when the patient is more stable, to reduce the burden of the study on the participants: *“bit more adaptable and you can speak to your doctor about what works best, and if phone appointments can be done”* and the possibility of spacing out appointments once the patient is more stable: *“I think that adding in how the patient would be contacted between, you know it says the amount of time between appointments would be increased or decreased based on how you’re doing.”* These useful suggestions from the CYP will be considered when designing the T2T trial. At the end of the animation, the CYP suggested that links to more information about the study should be included, in-case they had unanswered questions after viewing of the animation: *“Would put, especially for the older group, if there’s any place where you can get even more information, anyone they can ask or any links to a website”*. (See Fig. 1J). A summary of the main study findings is shown in Fig. 2, and a lay summary of the study is provided in the supplementary information (Additional File 1).

### Post-session survey

In the post-session survey, the participants understanding of T2T significantly increased to a median of 9 [IQR 8–10], *p* < 0.0001, on a 1–10 scale. When asked which methods the CYP thought would be best for explaining the concept of T2T, 28/37 (75.7%) responded that both an animation and written description would be best, 7/37 (18.9%) responded that an animation alone would be sufficient and 2/37 (5.4%) responded that they would prefer a written description of the study.

## Discussion

T2T is new concept in cSLE, and previous studies have found the concept difficult to understand amongst patients and parents [10]. This initiative had highlighted that CYP PPI groups can provide invaluable insights to improve communication with patients/parents about complex studies designs, and lead to important suggestions for improvement of the study design. Ideas and questions posed by children are significantly different from adults [24], therefore, their involvement enabled their ideas needs and preferences to be incorporated into the design of the resource. Many participants had characteristics of our intended target audience for the resources i.e. underlying rheumatic health condition and target age range, increasing the suitability of our resources for a cSLE trial [25].

The majority of CYP had not heard of T2T before, despite the use of T2T across different medical specialities. Lack of knowledge of the concept of T2T has previously been shown in RA patients. In-keeping with the results of the RA study, [17] viewing of a video resource significantly improved understanding of T2T, helping participants to consider T2T as an intervention.

CYP felt strongly that the use of phrases such as “if you aren’t meeting your target” should be removed from the animation, as they felt this placed blame on the patient for lack of progress. The CYP felt that this could impact upon the doctor-patient relationship and trust in their doctor, which could have a negative impact on patient satisfaction and medication compliance [26, 27]. Participants highlighted that the animation placed too much emphasis on the role of the computer, when making clinical decisions. They felt it needed to be stressed as an aid to the doctor, not a replacement.

The above issues were addressed in the revised storyboard, and resulted in changes to the illustrations and voiceover, explaining that the T2T paradigm does not replace the role of the doctor. In the updated storyboard, the doctor reviews the computer data and gives the patient a call to explain what is happening to emphasise that the human element of medicine remains. Previous studies have found that patients are more likely to follow a doctor’s recommendation for medical treatment, as compared to recommendations generated by a computer programme, and trust the doctor’s opinion more than a programme to make a good decision [28]. The adjustments to the storyboard demonstrate that the computer is there to support and not replace the doctor.

CYP felt that there should be less emphasis on parents during the conversations with the doctor about T2T. They explained that both clinical and research discussions during the consultation should be focused on the patient, with parents taking a back seat, but being present for support (where needed). The storyboard was therefore adjusted so the parents do not speak but are present. This was an important change, as allowing children to have increased autonomy and personal responsibility is key to successful transition and reducing the risk of parental overdependency and psychosocial delay, that children with chronic conditions are known to be at high risk of [29]. It is advised that children have the opportunity for “parent-free” consultations to help transition [30].

Although this PPI initiative aimed to generate feedback on resources to communicate T2T, the discussions also provided insight into the views of CYP on the concept of T2T. They highlighted the difficulty that families would have with increased frequency of T2T appointments (as compared to standard care), in-keeping with a quote from our recent qualitative study which explored the views of patients and parents on T2T: “*Families welcomed the increased frequency of hospital visits after the initial diagnosis. They suggested spacing visits out once things are more stable to reduce the impact on schooling, parental employment and family finances” *[10]*.* Poor school attendance is known to predispose CYP to emotional problems, negatively impacting upon the patients ability to cope with a chronic disease [31–33]. The PPI groups very astutely commented on this and provided practical suggestions as to how the study could be modified to reduce the impact on schooling and make the study more acceptable to children and families.

From the discussions with patients/parents included in our recent qualitative study [10], it was clear that targeting of disease activity alone is not sufficient and that families value a more holistic approach to treatment. In line with this, CYP participating in the PPI groups wanted to know if there would be other T2T treatments in addition to medicines. This led to incorporation of a physiotherapist, and specific mention of the fatigue and steroid toxicity measures in the updated storyboard.

We acknowledge important limitations in this work. Definite T2T targets and endpoints could not be explained within the animations as these are still being decided upon by a cSLE International T2T taskforce, through a series of international consensus meetings [34]. The level of understanding of the PPI group participants who viewed the animation and also participated in structured discussion about the animation, could be higher than for a patient who has never been involved in research before and only sees the animation once. It would therefore be important that the animation is easily accessible for potential trial participants, to enable repeated viewing of the animation if required. Due to the limited time available with the groups, we did not provide a hard copy of the voiceover to the participants or go through the script word-for-word. If more time had been available this is something we would have liked to do.

## Conclusion

This manuscript demonstrates an approach to involvement of CYP in the development of an audio-visual resource (animation) that will be integral to a future clinical trial, helping to describe the T2T approach in a way that makes sense to eligible CYC and their families. The resources will be of clear benefit in a future cSLE T2T study, supporting study recruitment. Involvement of CYP in research is crucial to help improve the design and delivery of studies, ensuring that they are relevant to CYP and their families. The initiative has also highlighted a range CYP’s view on the concept of T2T, with suggestions to improve the acceptability of such a study amongst CYP and their families. When developing the definitive trial protocol, further PPI involvement will be crucial.

## Supplementary Information

**Additional file 1.** Lay summary of the study.

## Data Availability

The datasets used and/or analysed during the current study are available from the corresponding author on reasonable request.

## Abbreviations

cSLE: Childhood onset systemic lupus erythematosus
CYP: Children and young people
HRQOL: Health related quality of life
JIA: Juvenile idiopathic arthritis
JSLE: Juvenile onset systemic lupus erythematous
MCTD: Mixed connective tissue disease
NRES: National Research Ethics Service
PPI: Public and patient involvement
T2T: Treat to target
TARGET LUPUS: Targeting disease, agreeing recommendations and reducing glucocorticoids through effective treatment in LUPUS
YPAG: Young Persons Advisory Group
