# Supplementary material for: Improving communication of the concept of 'treat-to target' in childhood lupus: a public and patient (PPI) engagement project involving children and young people
Source: BMC Rheumatol. 2022 Oct 15;6:69. doi: 10.1186/s41927-022-00300-z (PMC9578343; doi:10.1186/s41927-022-00300-z)
Supplement: Supplementary file 1 — Additional file 1. Lay summary of the study. [file 41927_2022_300_MOESM1_ESM.docx]

Treat-to-target (T2T) is a new method of treating Lupus, that will be studied in a future clinical trial. T2T is different from the way that Lupus patients are normally treated. A T2T approach is where treatment is intensified until a certain ‘target’ is reached, and re-intensified if the target is lost. Previous studies involving children and young people have shown that the idea of T2T can be difficult to understand. This paper describes a public and patient involvement (PPI) project, which aimed to develop an animation to explain the idea of T2T to children and families. We developed a PowerPoint presentation and showed this to young people attending three current PPI groups (called GenerationR, Lupus UK, and YOUR RHEUM), whilst at the same time performing a ‘voiceover’ to act out how the animation would come across. The PPI groups provided feedback, and made suggestions on how to improve the PowerPoint presentation and voiceover. Participants completed anonymous before/after workshop questionnaires, to test how well they understood the idea of T2T. 40 children and young people were involved overall, aged between 12 to 30 years old. Two thirds of the participants had an underlying rheumatic condition (e.g. Lupus, Arthritis). At the beginning of the workshop, the average understanding of T2T on a 1-10 scale (1 = “no understanding at all”, 10 = “completely confident in my understanding”) was 2 out of 10. After seeing the PowerPoint presentation and voiceover, the participant understanding improved to 9 out of 10. Overall, the participants felt that the mock up animation really improved their understanding of the idea of T2T. The children and young people made lots of useful suggestions for improvement of the future animation. Overall, this project has shown that involvement of children and young people in helping to design and plan research is of huge benefit. The final animation will be vital to a future clinical study, helping children and families to decide if they would like to be part of the T2T study.
